# Investigation of cardiopulmonary exercise testing using a dynamic leg press and comparison with a cycle ergometer

**DOI:** 10.1186/s13102-018-0095-3

**Published:** 2018-02-15

**Authors:** Farouk Chrif, Tobias Nef, Kenneth J. Hunt

**Affiliations:** 1 Institute for Rehabilitation and Performance Technology, Division of Mechanical Engineering, Department of Engineering and Information Technology, Bern University of Applied Sciences, Burgdorf, CH-3400 Switzerland; 2 Gerontechnology and Rehabilitation Research Group, ARTORG Center for Biomedical Engineering Research, University of Bern, Bern, CH-3008 Switzerland

**Keywords:** Cardiopulmonary exercise testing, Dynamic leg press, Cycle ergometer, Oxygen uptake, Heart rate, Ventilatory threshold

## Abstract

**Background:**

Leg-press machines are widely employed for musculoskeletal conditioning of the lower-limbs and they provide cardiovascular benefits for resistance training in cardiac patients. The aim of this study was to assess the feasibility of a dynamic leg press (DLP) for incremental cardiopulmonary exercise testing (CPET) and to compare the results with those obtained using a cycle ergometer (CE).

**Methods:**

Twelve healthy participants aged 27±4 years (mean ± standard deviation) performed incremental cardiopulmonary exercise tests on a DLP and on a CE. To facilitate CPET, the DLP was augmented with force and angle sensors, a work rate estimation algorithm, and a visual feedback system. Gas exchange variables and heart rate were recorded breath-by-breath using a cardiopulmonary monitoring system.

**Results:**

Peak oxygen uptake and peak heart rate were significantly lower for the DLP than for the CE: peak oxygen uptake was 3.2±0.5 vs. 4.1±0.5 L/min (DLP vs. CE, *p*=6.7×10^−6^); peak heart rate was 174±14 vs. 182±13 bpm (DLP vs. CE, *p*=0.0016). Likewise, the sub-maximal cardiopulmonary parameters, viz. the first and second ventilatory thresholds, and ramp duration were significantly lower for the DLP.

**Conclusions:**

The dynamic leg press was found to be feasible for CPET: the approach was technically implementable and all peak and sub-maximal cardiopulmonary parameters were able to be identified. The lower outcome values observed with the DLP can be attributed to a peripheral factor, namely the earlier onset of muscular fatigue.

## Background

Peak aerobic capacity in humans can be estimated using the highest value of the rate of oxygen uptake ($\dot {V}\text {O}_{2}$) obtained from incremental cardiopulmonary exercise testing [1–3]. Peak oxygen uptake, denoted $\dot {V}\text {O}_{2peak}$, is widely regarded as the gold standard measure for aerobic capacity [4, 5]. Estimation of peak aerobic capacity is important because it can be used not only for fitness assessment, but also for exercise intensity specification and prescription both in healthy individuals [6] and in patients [4, 7].

Cardiopulmonary exercise testing (CPET) is commonly administered using treadmills or cycle ergometers (CEs). Current cardiopulmonary exercise testing guidelines are based on standard exercise devices, namely treadmills and cycle ergometers (CEs) [4]. Any proposal for testing using new or modified devices should include a comparative assessment using one of these standard devices. In this vein, Orr et al. [8] compared the cardiopulmonary outcomes of an arm-crank device with those obtained with a cycle ergometer. They suggested the use of arm-crank cardiopulmonary exercise testing in those unable to cycle. Saengsuwan et al. [9] compared peak cardiopulmonary performance parameters from a robotics-assisted tilt table with both a treadmill and cycle ergometer. They demonstrated that the robotic tilt table is a valid and reliable device for CPET, and provides an alternative to the cycle ergometer and treadmill for the estimation of $\dot {V}\text {O}_{2\text {peak}}$ in severely impaired people who cannot use the standard modalities.

Leg-press machines are widely employed for musculoskeletal conditioning of the lower-limbs [10, 11]. They have also been demonstrated to provide cardiovascular benefits when employed for resistance training in cardiac patients [12]. Cardiopulmonary responsiveness, and in particular the applicability of such machines for estimation of peak and sub-maximal cardiopulmonary performance parameters during formal CPET, has not hitherto been investigated. The class of device termed “dynamic leg press” (DLP) is of particular interest for CPET because the forces acting on the footplates can be pre-programmed and continuously adjusted in a flexible manner.

Investigation of CPET using dynamic leg press exercise devices is important because formal CPET should ideally be conducted using a modality specific to the type of exercise training being carried out. That is to say, for cycle training CPET should be done on a cycle ergometer and for running training CPET should be done on a treadmill. In a similar vein, a person who is carrying out a training programme on a DLP should ideally be subjected to CPET using a DLP. The new methodology presented herein provides a means of doing CPET on the DLP, thus providing the specificity required for persons training on a DLP. Furthermore, the ability to employ DLPs in this manner would provide a complement to their application for musculoskeletal training.

A previous study that implemented a training programme using a leg-press machine, and which evaluated changes in peak $\dot {V}\text {O}_{2}$ using the leg press, a cycle, and a treadmill, found substantially and significantly larger increases when testing using the leg press in comparison with cycle and treadmill tests [13]. This underlines the importance of specificity of training and testing modalities.

The aim of this study was to assess the feasibility of a dynamic leg press for incremental cardiopulmonary exercise testing and to compare the results with those obtained using a standard exercise testing modality, viz. a cycle ergometer.

## Methods

### Participants and study design

This feasibility study was reviewed and approved by the Ethics Review Board of the Canton of Bern in Switzerland (Kantonale Ethikkommisssion Bern, KEK; Ref.: Basec-Nr. 2016-01502). Written informed consent was obtained from all participants prior to participation.

Twelve healthy male participants (age 27.0 ± 4.0 years, body mass 78.1 ± 5.7 kg) were recruited for participation in the study. To preserve homogeneity, inclusion criteria specified males aged 18–35 years who are regular exercisers (at least 3 times/week and 30 min/session). Smokers and persons with any prior history of cardiovascular or respiratory disease or with current musculoskeletal complaints or injuries were excluded.

Each participant performed two formal incremental cardiopulmonary exercise tests to their limit of tolerance, one on a dynamic leg press (DLP) and one on a cycle ergometer (CE). The tests were separated by at least 48 h [14, 15]. Prior to formal testing, and on a separate day, each participant attended a familiarisation session to be acquainted with the cardiopulmonary measurement equipment and with both exercise testing devices. During the familiarisation, participants carried out a 5-min bout of moderate-intensity exercise on both the DLP and the CE while wearing the cardiopulmonary monitoring devices (“Equipment” section, below). Participants were required to avoid strenuous activity within the 24 h prior to each formal test session, to refrain from caffeine for 12 h before, and not to consume a large meal within 3 h prior to testing.

**Fig. 1:**
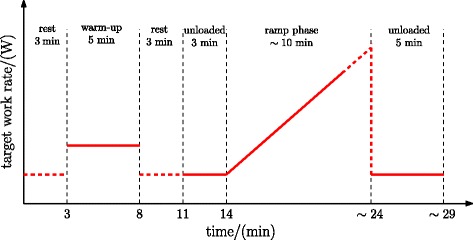
Exercise testing protocol

For both exercise devices, i.e. for the DLP and CE, formal peak-performance tests had six stages (Fig. 1): a 3-min recorded rest phase where the participant sat quietly on the exercise device; a 5-min warm up at low intensity (DLP - freely-chosen cadence, minimum force of 150 N; CE - unloaded cycling at self-selected cadence); a further 3 min of recorded rest; three minutes of low-intensity exercise as described above; a ramp phase of approximately ten minutes duration, where work rate increased linearly until the participant’s limit of exercise tolerance was reached; and a 5-min cool down exercising at low intensity. The transition between the first five stages of each test took place according to the fixed time intervals indicated in Fig. 1. Transition to the sixth stage was according to the participant’s volition: the primary end point for all tests was the participant’s own perception of having reached his peak exertion; the reason given by the participant for test termination was noted.

The tests were carried out using a counterbalanced design: the order of presentation of each test condition for each participant was sequentially changed, i.e. DLP then CE vs. CE then DLP, and by random assignment of participants upon recruitment. Thus, of the 12 participants, 6 were tested in the order DLP-CE and 6 in the order CE-DLP. Feasibility was assessed using the criteria of technical implementability and cardiopulmonary responsiveness.

### Equipment

**Fig. 2:**
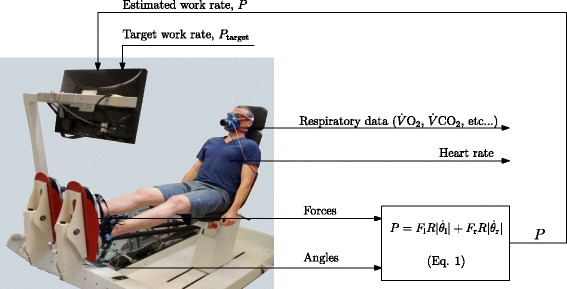
Dynamic leg press augmented with work rate estimation and visual feedback

A commercial pneumatically-actuated dynamic leg press was employed (Allegro, Dynamic Devices AG, Switzerland). For the comparative evaluation, a cycle ergometer was used (model LC7, Monark Exercise AB, Sweden). To facilitate CPET, the DLP was augmented with force sensors in the footplates, angle sensors at the rotation axes of the pedals, a work rate estimation algorithm, and a visual feedback system (“Work rate estimation and control” section, below, and Fig. 2).

Respiratory variables and heart rate were recorded using a breath-by-breath cardiopulmonary monitoring system (Metamax 3B, Cortex Biophysik GmbH, Germany; Fig. 2). Analysis of the cardiopulmonary data was done using the proprietary software associated with the breath-by-breath system (Metasoft, version 3.9.9 SR5). Pressure, volume and gas concentrations were calibrated prior to each test according the manufacturer’s instructions: pressure was calibrated using a certified atmospheric pressure device; volume using a 3 L syringe; and gas concentrations were calibrated using ambient air and a precision gas mixture (15% O_2_, 5% CO_2_). Heart rate was monitored using a chest belt (T34, Polar Electro Oy, Finland).

### Work rate estimation and control

On the DLP, the participant’s feet were fixed on footplates within which force sensors were mounted. The position and angular velocity of the pedals were obtained using angle sensors mounted at the rotation axis of each pedal. The participant’s total work rate (*P*) was estimated using the force and velocity data as follows:

1 $$  \begin{aligned} P & = P_{\mathrm{l}} + P_{\mathrm{r}} \\ & = F_{\mathrm{l}} R \lvert \dot{\theta}_{\mathrm{l}} \lvert + F_{\mathrm{r}} R \lvert \dot{\theta}_{\mathrm{r}} \lvert \\ & = R(F_{\mathrm{l}} \lvert \dot{\theta}_{\mathrm{l}} \lvert + F_{\mathrm{r}} \lvert \dot{\theta}_{\mathrm{r}} \lvert). \end{aligned}  $$

In the above equation, *P*_l_ and *P*_r_ are the work rates of the left and right legs, *F*_l_ and *F*_r_ are the forces applied by the left and right legs, *R* is the radius of the pedal motion (distance between the footplate and the rotation axis), and $\dot {\theta }_{\mathrm {l}}$ and $\dot {\theta }_{\mathrm {r}}$ are the left and right angular velocities. Since the DLP exercise involves both positive and negative work, depending on the movement direction, the modulus operator is employed above to give the total mechanical work rate of the participants.

During the ramp phase of each test, the left and right forces *F*_l_ and *F*_r_ were set to be equal. In the first 3 min of the ramp phase, the left and right forces were constant (150 N); after the third minute the forces started to increase linearly with time, with a slope of 16.7 N/min.

During all tests, for both the DLP and the CE, participants were instructed to maintain a constant exercise cadence of 60 cycles/min. This was achieved on the DLP using an electronic metronome, and on the CE using a numerical display.

For the DLP, the estimated work rate was displayed continuously on a visual feedback screen together with an individualised target work rate (*P*_target_), the latter following the profile defined in the incremental test protocol (Fig. 1). participants were required to keep the estimated work rate (*P*) as close as possible to the target work rate (*P*_target_) by adapting their volitional effort (Fig. 2). The above work rate estimation algorithm was implemented in real-time in the Matlab/Simulink environment (Mathworks Inc., USA).

As a consequence of the linearly increasing target work rate and the imposition of a constant cadence, participants were instructed to adopt the strategy of gradually increasing the stroke of their leg extensions/flexions during the ramp phase. This in turn resulted in increasing angular velocities (since, as a consequence of the constant cadence, a longer distance was covered in the same time) and to the desired increase in work rate, Eq. (1).

On the CE, pedalling resistance was automatically adjusted by the CE’s internal software to achieve the target work rate according to the test profile (Fig. 1). The target work rate profile was implemented in the Metasoft software running on a PC, which communicated with the CE in real-time during each test.

For the CE, the slope of the target work rate during the incremental phase of each test was set individually for each participant using a method for predicting peak work rate that is documented elsewhere [16]. The prediction algorithm uses the participant’s age, body mass and exercise habits. The incremental work rate slope was then set in order to reach the predicted peak work rate in 10 min [1].

The individual predicted peak work rate for the DLP was modified in consideration of the fact that exercise on the DLP consists of both concentric (positive) and eccentric (negative) muscular work (on the CE, the work is only concentric). Since the metabolic cost of negative work is approximately one-third of that for concentric work [17], a higher external work rate would be expected at the limit of exercise tolerance, wherefore the target peak work rate for the DLP was chosen to be higher by a factor of 1.4 than the individual participant’s value estimated for the CE. This factor was obtained as an estimate based on a series of pilot measurements, and based on the above consideration of the relative metabolic cost of concentric (positive) and eccentric (negative) work.

### Outcome measures

Six outcome measures were estimated for each test. These comprised three peak cardiopulmonary performance parameters, two sub-maximal thresholds and ramp duration [1]. The three peak-performance outcomes were:

- Peak oxygen uptake, denoted $\dot {V}\text {O}_{2\text {peak}}$, taken to be the highest value of $\dot {V}\text {O}_{2}$ from a 15-breath moving average.
- Peak heart rate, HR_peak_.
- Peak respiratory exchange ratio, RER_peak_, the 15-breath moving average value of RER at the time of $\dot {V}\text {O}_{2\text {peak}}$. RER is given by $\text {RER} = \dot {V}\text {CO}_{2}/\dot {V}\text {O}_{2}$.

The two sub-maximal outcomes were the oxygen uptake at the first and second ventilatory thresholds, denoted $\dot {V}\text {O}_{\mathrm {2VT1}}$ and $\dot {V}\text {O}_{\mathrm {2VT2}}$. The VTs were determined according the criteria documented in Binder et al. [18]:

- Oxygen uptake at the first ventilatory threshold, $\dot {V}\text {O}_{\mathrm {2VT1}}$, was determined by:
  1. calculation of the point of deflection of $\dot {V}\text {CO}_{2}$ versus $\dot {V}\text {O}_{2}$ (V-slope method);
  2. visual inspection of the point where $\dot {V}\mathrm {E}/\dot {V}\text {O}_{2}$ reaches its minimum or starts to rise without a rise in $\dot {V}\mathrm {E}/\dot {V}\text {CO}_{2}$; and,
  3. visual inspection of the point at which partial pressure of end-tidal oxygen tension (P _ET_O_2_) reaches a minimum or starts to rise without a decline in the partial pressure of end-tidal carbon dioxide tension (P _ET_CO_2_).
- Oxygen uptake at the second ventilatory threshold, $\dot {V}\text {O}_{\mathrm {2VT2}}$, was determined by:
  1. calculation of the point of deflection of $\dot {V}\mathrm {E}$ versus $\dot {V}\text {CO}_{2}$;
  2. visual inspection of the point where $\dot {V}\mathrm {E}/\dot {V}\text {CO}_{2}$ reaches its minimum or starts to increase non-linearly; and,
  3. visual inspection of the point where P _ET_CO_2_ starts to decline.

These criteria were applied independently by two experienced raters (authors FC and KJH); any discrepancies were then resolved by mutual agreement.

The duration of the ramp phase (Fig. 1), denoted *t*_ramp_, was also recorded. *t*_ramp_ is defined as the duration between ramp onset and the time of $\dot {V}\text {O}_{\mathrm {2peak}}$.

### Statistical analysis

To compare test results between the DLP and CE, comparison of means was carried out for all six outcomes. Outcome differences were checked for normality using the Kolomogorov-Simirnov test with Lilliefors correction, and paired-sample two-sided t-tests were applied (all data were found to be normal). The null hypothesis for each comparison was that there is no difference between the DLP and the CE, and the significance level was set as *α*=0.05. The relationships between the DLP and CE outcomes were assessed using linear regression correlation analysis. All analyses were performed using the Matlab Statistics and Machine Learning Toolbox (MathWorks Inc., USA).

## Results

**Fig. 3:**
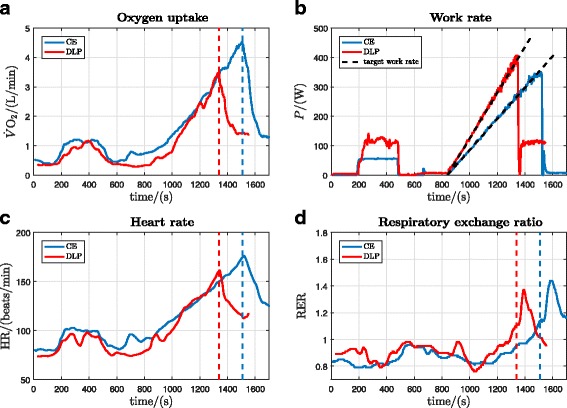
Original data records from one participant’s exercise tests on the DLP and CE (participant 8). **a** Oxygen uptake ($\dot {V}\text {O}_{2}$). **b** Target and measured work rates (*P*_target_, *P*). **c** Heart rate (HR). **d** Respiratory exchange ratio (RER)

**Fig. 4:**
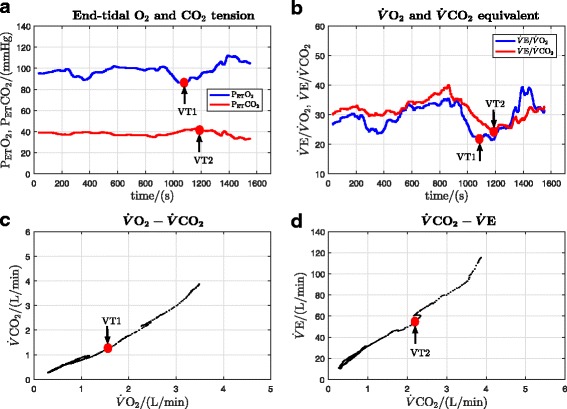
Original data record and determination of VT1 and VT2 thresholds for the DLP data in Fig. 3 (participant 8). **a** VT1 is at the minimum point of P _ET_O_2_ and VT2 is at the turning point of P _ET_CO_2_. **b** VT1 is at the minimum point of $\dot {V}\mathrm {E}/\dot {V}\text {O}_{2}$ and VT2 is the minimum point of $\dot {V}\mathrm {E}/\dot {V}\text {CO}_{2}$. **c** VT1 is at the deflection point of $\dot {V}\text {CO}_{2}$ vs. $\dot {V}\text {O}_{2}$ (V-slope method). **d** VT2 is at the deflection point of $\dot {V}\mathrm {E}$ vs. $\dot {V}\text {CO}_{2}$

**Fig. 5:**
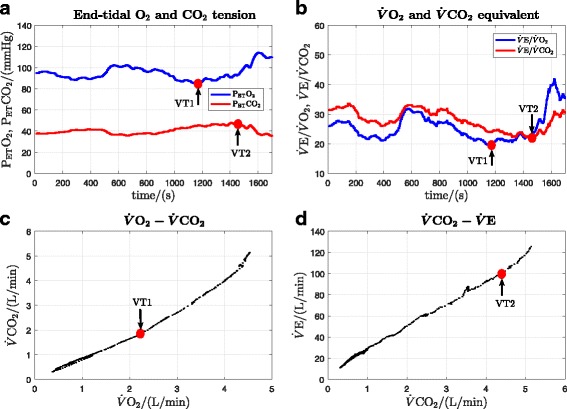
Original data record and determination of VT1 and VT2 thresholds for the CE data in Fig. 3 (participant 8). **a** VT1 is at the minimum point of P _ET_O_2_ and VT2 is at the turning point of P _ET_CO_2_. **b** VT1 is at the minimum point of $\dot {V}\mathrm {E}/\dot {V}\text {O}_{2}$ and VT2 is the minimum point of $\dot {V}\mathrm {E}/\dot {V}\text {CO}_{2}$. **c** VT1 is at the deflection point of $\dot {V}\text {CO}_{2}$ vs. $\dot {V}\text {O}_{2}$ (V-slope method). **d** VT2 is at the deflection point of $\dot {V}\mathrm {E}$ vs. $\dot {V}\text {CO}_{2}$

In order to illustrate the method of calculation of the primary outcomes, original data records for a single participant (participant 8) are presented. These records show: oxygen uptake, heart rate, work rate and RER for both the DLP and CE (Fig. 3); determination of VT1 and VT2 for the DLP (Fig. 4); and determination of VT1 and VT2 for the CE (Fig. 5). All cardiopulmonary outcome measures could be successfully estimated for all participants for both the DLP and the CE, except that VT2 could not be identified for two participants on the DLP.

A summary of the overall statistical analysis of DLP vs. CE outcomes across all participants is provided (Table 1): the mean values of five of the six outcomes were significantly lower for the DLP than for the CE; only RER_peak_ did not show a significant difference. All outcomes for each individual participant are reported in Table 2.

**Table 1:** Outcome values from the DLP and CE

	mean ± SD		MD (95% CI)	*p*-value
	DLP	CE	DLP - CE	
$\dot {V}\text {O}_{\mathrm {2peak}}$/(L/min)	3.207 ±0.499	4.099 ±0.492	-0.892 (-1.138,-0.646)	0.0000067
HR_peak_/(bpm)	174 ±14	182 ±13	-8 (-12,-4)	0.0016
RER_peak_	1.21 ±0.07	1.18 ±0.05	0.02 (-0.01,0.06)	0.17
$\dot {V}\text {O}_{\mathrm {2VT1}}$/(L/min)	1.555 ±0.358	1.779 ±0.349	-0.224 (-0.421,-0.027)	0.029
$\dot {V}\text {O}_{\mathrm {2VT2}}$/(L/min)	2.704 ±0.414	3.472 ±0.551	-0.768 (-1.140,-0.396)	0.0012
*t*_ramp_/(min)	9.3 ±1.5	10.7 ±1.2	-1.4 (-2.1,-0.8)	0.00067

*n*=12, except $\dot {V}\text {O}_{\mathrm {2VT2}}$ (*n*=10)

DLP: dynamic leg press

CE: cycle ergometer

MD: mean difference of DLP - CE

SD: standard deviation

95% CI: 95% confidence interval for the mean difference

*p*-values are: paired two-sided t-tests

**Table 2:** Participants’ individual cardiopulmonary outcomes

	DLP					CE				
Participant	$\dot {V}\text {O}_{\mathrm {2peak}}$	HR_peak_	RER	$\dot {V}\text {O}_{\mathrm {2VT1}}$	$\dot {V}\text {O}_{\mathrm {2VT2}}$	$\dot {V}\text {O}_{\mathrm {2peak}}$	HR_peak_	RER	$\dot {V}\text {O}_{\mathrm {2VT1}}$	$\dot {V}\text {O}_{\mathrm {2VT2}}$
1	3.436 (40.0)	178	1.17	1.641 (19.1)	2.415 (28.1)	4.186 (48.7)	176	1.17	2.225 (25.9)	3.511 (40.8)
2	2.786 (39.8)	192	1.19	1.048 (15.0)	2.321 (33.2)	3.313 (47.3)	195	1.25	1.512 (21.6)	3.144 (44.9)
3	3.710 (44.2)	151	1.16	1.133 (13.5)	2.874 (34.2)	4.247 (50.6)	161	1.11	1.702 (20.3)	2.943 (35.0)
4	3.318 (41.0)	193	1.19	1.513 (18.7)	2.517 (31.1)	4.174 (51.5)	199	1.23	1.534 (18.9)	3.151 (38.9)
5	4.217 (57.8)	188	1.20	2.258 (30.9)	3.636 (49.8)	5.145 (70.5)	195	1.21	2.299 (31.5)	4.452 (61.0)
6	2.905 (41.5)	168	1.21	2.146 (30.7)	-	4.234 (60.5)	182	1.19	2.023 (28.9)	-
7	2.507 (34.3)	180	1.24	1.487 (20.4)	-	3.638 (49.8)	183	1.12	1.150 (15.8)	-
8	3.470 (45.7)	171	1.25	1.481 (19.5)	3.007 (39.6)	4.061 (53.4)	175	1.22	1.695 (22.3)	3.927 (51.7)
9	3.486 (43.6)	163	1.11	1.559 (19.5)	2.382 (29.8)	4.530 (56.6)	177	1.14	2.230 (27.9)	4.256 (53.2)
10	2.930 (38.1)	157	1.20	1.237 (16.1)	2.675 (34.7)	3.578 (46.5)	160	1.14	1.544 (20.1)	2.910 (37.8)
11	3.150 (38.0)	182	1.20	1.504 (18.1)	2.890 (34.8)	3.707 (44.7)	192	1.17	1.740 (21.0)	3.152 (38.0)
12	2.565 (30.5)	163	1.38	1.652 (19.7)	2.318 (27.6)	4.370 (52.0)	185	1.26	1.691 (20.1)	3.269 (38.9)
Mean ± SD	3.207 ±0.499	174 ±14	1.21 ±0.07	1.555 ±0.358	2.704 ±0.414	4.099 ±0.492	182 ±13	1.18 ±0.05	1.779 ±0.349	3.472 ±0.551
	(41.2 ±6.7)			(20.1 ±5.4)	(34.3 ±6.5)	(52.7 ±7.1)			(22.8 ±4.7)	(44.0 ±8.5)

*n*=12, except $\dot {V}\text {O}_{\mathrm {2VT2}}$ (*n*=10)

$\dot {V}\text {O}_{2}$ related measures in L/min (mL/kg/min)

HR in bpm

**Fig. 6:**
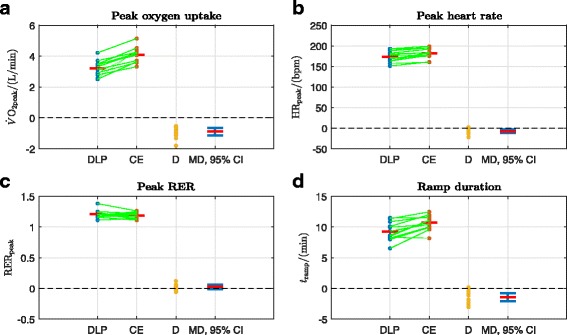
Peak performance parameters and ramp duration. the green lines link the sample pairs from each participant; the red horizontal bars depict mean values. D is the difference between the paired samples: D = DLP - CE. MD is the mean difference (red horizontal bar), with its 95% confidence interval (CI) in blue. Inclusion of the value 0 within the 95% CI signifies a non-significant difference between the means (*p*>0.05, Table 1); a significant difference between the means is marked by 0 lying outwith the 95% CI (*p*<0.05, Table 1). **a**$\dot {V}\text {O}_{\mathrm {2peak}}$: MD = -0.892 L/min, 95% CI = (-1.138,-0.646), *p*=0.0000067. **b** HR_peak_: MD = -8 bpm, 95% CI = (-12,-4), *p*=0.0016. **c** RER_peak_: MD = 0.02, 95% CI = (-0.01,0.06), *p*=0.17. **d** *t*_ramp_: MD = -1.4 min, 95% CI = (-2.1,-0.8), *p*=0.00067

$\dot {V}\text {O}_{\mathrm {2peak}}$ was lower on the DLP (3.207±0.499 L/min) (mean ± SD) compared to the CE (4.099±0.492 L/min), (*p*=0.0000067, Table 1, Fig. 6a). HR_peak_ was 174±14 bpm vs. 182±13 bpm, DLP vs. CE (*p*=0.0016, Table 1, Fig. 6b). There was no significant difference in RER_peak_: 1.21±0.07 vs. 1.18±0.05, DLP vs. CE (*p*=0.17, Table 1, Fig. 6c).

**Fig. 7:**
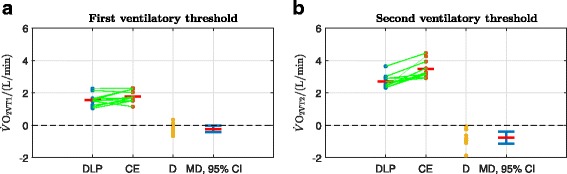
Sub-maximal outcomes: the green lines link the sample pairs from each participant. the red horizontal bars depict mean values. D is the difference between the paired samples: D = DLP - CE. MD is the mean difference (red horizontal bar), with its 95% confidence interval (CI) in blue. A significant difference between the means is marked by 0 lying outwith the 95% CI (*p*<0.05, Table 1). **a**$\dot {V}\text {O}_{\mathrm {2VT1}}$: MD = -0.224 L/min, 95% CI = (-0.421,-0.027), *p*=0.029. **b**$\dot {V}\text {O}_{\mathrm {2VT2}}$: MD = -0.768 L/min, 95% CI = (-1.140,-0.396), *p*=0.0012

The first ventilatory threshold, VT1, was able to be identified for all 12 participants on both devices. $\dot {V}\text {O}_{\mathrm {2VT1}}$ was 1.555±0.358 L/min vs. 1.779±0.349 L/min, DLP vs. CE (*p*=0.029, Table 1, Fig. 7a). The second ventilatory threshold, VT2, could not be identified for 2 participants on the DLP. For *n*=10, $\dot {V}\text {O}_{\mathrm {2VT2}}$ was 2.704±0.414 L/min vs. 3.472±0.551 L/min, DLP vs. CE (*p*=0.0012, Table 1, Fig. 7b).

The ramp duration for the DLP was significantly shorter than for the CE: 9.3±1.5 min vs. 10.7±1.2 min, DLP vs. CE (*p*=0.00067, Table 1, Fig. 6d). Ramp duration for the CE was between 8 min 9 s and 12 min 28 s, which is within the recommended time range for incremental CE exercise testing of 7–26 min [19]. For the DLP, the observed ramp duration was from 6 min 30 s to 11 min 30 s.

The linear-regression analysis for the cardiopulmonary outcomes (DLP vs. CE) gave correlation coefficients in the range 0.45 – 0.88 (moderate to strong correlations, Table 3).

**Table 3:** Linear regression (correlation) analysis: DLP vs. CE

		*r*	*p*-value	95% CI		SEE
HR_peak_		0.88	0.00015	0.62,0.97		7
$\dot {V}\text {O}_{\mathrm {2peak}}$		0.69	0.012	0.20,0.91		0.376
RER		0.53	0.076	-0.06,0.85		0.06
$\dot {V}\text {O}_{\mathrm {2VT1}}$		0.62	0.033	0.06,0.88		0.296
$\dot {V}\text {O}_{\mathrm {2VT2}}$		0.45	0.20	-0.25,0.84		0.393
*t*_ramp_		0.73	0.0067	0.26,0.84		1.1

*n*=12, except $\dot {V}\text {O}_{\mathrm {2VT2}}$ (*n*=10)

*r*: correlation coefficient

95% CI: 95% confidence interval for the correlation coefficient

SEE: standard error of estimate

For units of SEE, see Table 1

## Discussion

The aim of this study was to assess the feasibility of a dynamic leg press for incremental cardiopulmonary exercise testing and to compare the results with those obtained using a standard exercise testing modality (a cycle ergometer).

The results showed that it is feasible to employ the DLP for incremental CPET, both in terms of technical implementability and cardiopulmonary responsiveness. To facilitate CPET, the DLP used in this study was augmented with force and angle sensors, a work rate estimation algorithm, and a visual feedback system. This allowed specific work-rate profiles to be imposed upon the participants; using the visual feedback system, all participants were able to follow the target work rate accurately.

The substantial cardiopulmonary responses observed with the DLP allowed all of the peak and sub-maximal cardiopulmonary response parameters to be identified with a high rate of success; in just two of the twelve participants, the VT2 threshold could not be clearly identified. This is likely to have been because these participants terminated the exercise before the VT2 was reached due to muscular fatigue and/or discomfort (the VT2 is commonly also referred to as the respiratory compensation point (RCP), and occurs at very high exercise intensity of up to 90% of $\dot {V}\text {O}_{\mathrm {2peak}}$ [18]): the reason given by all participants for test termination on the DLP was leg-muscle fatigue and/or discomfort; for the CE, the reason given by 9 participants was that their cardiorespiratory limit had been reached, while for 3 participants the stopping reason was both cardiorespiratory limitation and muscle fatigue.

It was observed that all outcomes, with the exception of RER_peak_, were significantly lower for the DLP than for the CE. This is likely to be related to the fact noted above that, on the DLP, test termination was due to muscular factors. In contrast, CE tests were all terminated due to the limit of cardiorespiratory function having been reached. The involvement of peripheral rather than central mechanisms in test termination thus led to the observation of significantly lower $\dot {V}\text {O}_{\mathrm {2peak}}$ and HR_peak_ on the DLP, and also to significantly lower sub-maximal thresholds VT1 and VT2.

These factors are due in turn to differences in the nature of the exercise and muscular work performed on the DLP and on the CE. On the DLP, forces experienced by the participants at the footplates have to be actively resisted during both the extension and flexion phases of leg motion. This means that both concentric and eccentric muscle contractions have to take place, and that the muscles are continuously active [13, 20, 21]. In contrast, on the CE the muscular work is concentric only and the muscles have a period of rest during the flexion phase of each leg cycle [13, 22]. The fact that the legs have no rest phase during leg-press exercise was noted in a previous study to be likely to alter venous return and limit stroke volume (SV), thus contributing to the lower peak cardiopulmonary outcomes [13].

Despite these differences in the reasons for test termination, and the lower outcomes observed for the DLP, both modes of exercise displayed similar and high values of RER_peak_ (mean values: 1.21 for DLP, 1.18 for CE; Table 1) thus fulfilling one of the recommended criteria for confirmation of a maximal response, which include a value for $\text {RER}_{\text {peak}} \geqslant 1.10$ [5].

Differences in peak and sub-maximal cardiopulmonary outcomes have been observed in other studies which compared different modes of exercise in a single participant cohort. It was found that mean $\dot {V}\text {O}_{\mathrm {2peak}}$ for a CE was 12% lower than for the treadmill, and, in turn, that $\dot {V}\text {O}_{\mathrm {2peak}}$ for the robotics-assisted tilt table was 20% lower than for the CE [9, 23]. In the present study, mean $\dot {V}\text {O}_{\mathrm {2peak}}$ for the DLP was 22% lower than for the CE (3.2 vs. 4.1 L/min, Table 1). There are parallels between the outcomes of these two studies in that the magnitude of reduction in $\dot {V}\text {O}_{\mathrm {2peak}}$ for the new device under assessment was similar, and in that the leg motion for the robotics-assisted tilt table was also found to be inefficient in terms of cardiopulmonary forcing [9].

As noted above, the semi-recumbent, seated position of people using the DLP, with the feet secured safely in the footplates, may in certain target populations with neurological deficits (e.g. stroke) have advantages compared to treadmills or upright cycle ergometers. An alternative modality for safely investigating cardiopulmonary outcomes in impaired participants is the arm ergometer [8, 24, 25]. In healthy participants, $\dot {V}\text {O}_{\mathrm {2peak}}$ obtained from an arm ergometer was 30–34% lower than for a cycle ergometer [8, 26]. It would therefore be of interest to investigate whether there are any differences in cardiopulmonary outcomes between the DLP and the arm ergometer.

Mean ramp duration was significantly shorter for the DLP than for the CE (9.3 vs. 10.7 min, Table 1). This result is in line with the differences in most cardiopulmonary outcomes discussed above. Contemporary guidelines for incremental CPET recommend a ramp duration of 5–26 min for a treadmill and 7–26 min for a CE (review: [19]). In the present study, all observed CE ramp durations fell within the recommended range for CEs. For the DLP, the ramp duration varied between 6 min 30 s and 11 min 30 s. Only one test (the test with duration 6 min 30 s) had a duration outside the recommended range for CEs of 7 to 26 min, but it was still within the recommended range for treadmills, which is 5 to 26 min.

Despite these considerations, the differences in ramp duration represent a limitation of the present study. Thus, further investigations specific to the DLP are warranted to establish an appropriate/optimal range for ramp duration. This question is linked to the need to establish an accurate method for prediction of peak work rate on the DLP. Here, a method developed for the CE was employed, [16], with adaptations to account for the presence of negative muscular work (“Methods” section). The factor used for scaling predicted peak work rate, selected here as 1.4, may have been overestimated since the mean ramp duration for the DLP of 9.3 min was lower than the target duration of 10 min. However, in view of the apparently rapid onset of peripheral fatigue, these investigations should remain open to the possibility that a relatively short ramp duration may be optimal for DLP-based testing; this would call for a peak-work-rate target duration substantially shorter than the 10 min employed here.

The feasibility of the new methodology presented herein is important for the DLP device because it provides the specificity of testing required for persons who are also training on a DLP. The fact that the DLP outcomes were found to be lower than the CE outcomes is not a decisive factor in the assessment of feasibility: it is known that different types of testing device can give substantially different levels of peak $\dot {V}\text {O}_{2}$ (e.g. outcomes on a CE are substantially lower than those obtained from a treadmill [27–29]).

The correlation analysis for the DLP vs. CE shows that there are moderate to strong linear correlations between the DLP and CE outcomes (Table 3). Since the CE is already established as a valid and reliable testing modality, the level of correlation gives a degree of evidence that the DLP outcomes are also valid.

As a complement to DLP-based CPET, future work should also focus on assessment of the DLP for cardiopulmonary exercise training. Moderate-intensity continuous training and high-intensity interval training approaches should be investigated [30, 31]. Further assessment is also necessary to assess the feasibility and clinical relevance of CPET using the DLP in different patient populations. Since the use of the DLP for CPET is a new method, test-retest reliability and repeatability should also be investigated in a separate study.

## Conclusions

The dynamic leg press was found to be feasible for incremental cardiopulmonary exercise testing: the approach was technically implementable and all peak and sub-maximal cardiopulmonary parameters were able to be identified. The lower outcome values observed with the DLP can be attributed to a peripheral factor, namely the earlier onset of muscular fatigue.

## Abbreviations

bpm: Beats per minute
CE: Cycle ergometer
CI: Confidence interval
CPET: Cardiopulmonary exercise testing
DLP: Dynamic leg press
HR: Heart rate
HR_peak_: Peak heart rate
MD: Mean difference
*P*: Work rate (synonymous with “power”)
P _ET_CO_2_: Partial pressure of end-tidal carbon dioxide tension
P _ET_O_2_: Partial pressure of end-tidal oxygen tension
*P*_target_: Target work rate
RER_peak_: Peak respiratory exchange ratio
RER: Respiratory exchange ratio, $\text {RER} \triangleq \dot {V}\text {CO}_{2}/\dot {V}\text {O}_{2}$
SD: Standard deviation
*t*_ramp_: Ramp duration
$\dot {V}\mathrm {E}$: Minute ventilation
$\dot {V}\mathrm {E}/\dot {V}\text {CO}_{2}$: Ventilatory equivalent of carbon dioxide
$\dot {V}\mathrm {E}/\dot {V}\text {O}_{2}$: Ventilatory equivalent of oxygen
$\dot {V}\text {CO}_{2}$: Rate of carbon dioxide output
$\dot {V}\text {O}_{2}$: Rate of oxygen uptake (referred to simply as “oxygen uptake”)
$\dot {V}\text {O}_{\mathrm {2peak}}$: Peak oxygen uptake
$\dot {V}\text {O}_{\mathrm {2VT1}}$: Oxygen uptake at the first ventilatory threshold
$\dot {V}\text {O}_{\mathrm {2VT2}}$: Oxygen uptake at the second ventilatory threshold
VT1: First ventilatory threshold
VT2: second ventilatory threshold
